# Knowledge of Knee Osteoarthritis and Its Impact on Health in the Middle East: Are They Different to Countries in the Developed World? A Qualitative Study

**DOI:** 10.1155/2020/9829825

**Published:** 2020-05-07

**Authors:** Lara Al-Khlaifat, Rasha Okasheh, Jennifer Muhaidat, Ziad M. Hawamdeh, Dania Qutishat, Emad Al-yahya, Jihad M. Al-ajlouni, Maha T. Mohammad

**Affiliations:** ^1^Physiotherapy Department, School of Rehabilitation Sciences, The University of Jordan, Amman 11942, Jordan; ^2^School of Medicine, The University of Jordan, Amman 11942, Jordan

## Abstract

Knowledge of knee osteoarthritis (OA) and its management options affects adherence to treatment, symptoms, and function. Many sociocultural differences exist between Jordan, as a representative of the Middle East, and the developed world which might influence the knowledge of the pathology and its impact on health. *Objectives*. To explore the knowledge of the pathology and the experience of people diagnosed with knee OA living in Jordan. *Methods*. Qualitative study design using a triangulation method of both focus groups and in-depth semistructured interviews. Fourteen participants were included (13 females and one male). One focus group and seven in-depth semistructured interviews were conducted. Discussions were audiotaped and transcribed. Framework analysis was used, and data were mapped to the International Classification of Functioning, Disability and Health framework. *Results*. The themes are as follows: (1) body functions and structures included two subthemes: physical changes and psychological impact; (2) activity limitation and participation restriction included three subthemes: factors influencing the activities, cultural and social perspectives to activity limitation, and participation restriction; (3) personal factors included three subthemes: knowledge and personal interpretation of disease process, knowledge of management options to relief symptoms, and influence of personal factors on activity and participation; and (4) environmental factors included three subthemes: service delivery process, ineffective communication across the care pathway, and facilitators and barriers. *Conclusions*. Knowledge of the disease was lacking as a consequence of inappropriate service delivery and culture. Activity limitations and participation restrictions are similar in Jordan to other cultures in addition to limitations in religious, employment, and transportation activities. The results demonstrate that the effect of knee OA varies among different cultures and highlight the role of healthcare professionals worldwide in understanding the impact of culture on health. They also increase the awareness of healthcare professionals, specifically in Jordan, on the limitations in delivered services and the importance of education.

## 1. Introduction

Knee osteoarthritis (OA) has a major effect on function and quality of life [1]. Lack of knowledge of knee OA and its management options decreases adherence to treatment and consequently affects symptoms and function and increases healthcare costs [2, 3]. People living mainly in the developed world identified many aspects to affect their experience with the disease including level of understanding of the pathology, its symptoms, how others perceive it, and functional impairment [4]. However, these aspects cannot express the experiences of people from different cultural backgrounds in the developing world such as the Middle East.

Knee OA is a common musculoskeletal problem in Jordan, one of the countries in the Middle East Region; however studies exploring its prevalence and risk factors are lacking. Hawamdeh and Al-Ajlouni [5] showed that Jordanians have higher severity of knee OA compared to the developed world, and they referred this to higher body mass index and cultural habits such as praying and cross-sitting.

The population is mainly Muslim, and those who practice Islam pray five times a day; this involves transitioning between heel sitting, prostration, and standing. The atmosphere and geography do not support outdoor physical activity and exercise; weather is hot in summer, proper infrastructure is lacking in winter, and straight roads are limited. Moreover, public transportation is not well-organized [6]. The culture mostly favors men over women as demonstrated by the Global Gender Index, e.g., women were mainly unemployed [7]. These sociocultural differences when compared to countries of the developed world might affect the impact of knee OA on health.

It is essential for healthcare professionals to understand the impact of culture in people with knee OA worldwide to modify treatment plans accordingly and educate people about the pathology and its management. These modifications would improve adherence to treatment which would improve the patient's experience with knee OA and decrease healthcare costs.

To understand the impact of knee OA on health worldwide, results could be mapped to international frameworks to allow for international comparison. The International Classification of Functioning, Disability and Health (ICF) framework was approved by the World Health Organization in 2001 [8]. It considers symptoms, limited activities, and restricted participation caused by any medical condition in addition to environmental and personal factors that could affect the patient.

Therefore, this study is aimed at exploring the knowledge of people diagnosed with knee OA living in Jordan of their condition and its management options, in addition to exploring the impact of knee OA on their health and mapping the results to the ICF framework.

## 2. Methods

### 2.1. Study Design

We adopted a qualitative study design based on the grounded theory of Strauss and Corbin [9]. We used the triangulation method for data collection by conducting both focus groups and in-depth semistructured interviews. We reported our procedure and results according to the Standards for Reporting Qualitative Research (SRQR) checklist [10] (Appendix 1).

### 2.2. Recruitment and Sample Size

People diagnosed with knee OA were recruited from orthopedic clinics and the rehabilitation department at a local hospital. Inclusion criteria included adults who speak Arabic, aged ≥40 years, and diagnosed with knee OA based on clinical and radiographic evaluation by their physicians and radiologist or from medical records. Exclusion criteria included diagnosis of any orthopedic (other than OA) or neurological condition confirmed by self-report or from medical records.

The principal investigator was the first to contact the participants. Those who met the inclusion criteria and consented to take part were contacted by phone to organize the date of focus group or interviews. Few female participants (*n* = 20) declined to participate, and all reported personal reasons including lack of time, cannot come alone, lives far away from the hospital, and not interested.

All potential male participants except for one refused to participate, and because of time restrictions to complete the study, we conducted one semistructured interview for him to explore his knowledge and experience in details, and no focus group with males was performed. Therefore, a convenient sample of fourteen participants was included in the study.

### 2.3. Procedure

This study was approved by the research ethics committee at Jordan University Hospital. All methods were carried out in compliance with the latest guidelines and regulations of the Declaration of Helsinki. All participants provided informed written consent prior to study commencement.

We started by conducting one focus group of seven female participants, as it is suggested to include between six and eight participants in each focus group [11], to encourage discussions and enrich emerging data [12]. To gain a deeper understanding of their experience, we conducted additional in-depth semistructured interviews with females until data saturation was achieved after the 7th interview where new information did not emerge [13, 14].

We followed recommended procedures for focus groups and semistructured interviews [11, 15]. Participants came once to the meeting room at the physiotherapy department at a main local university for a session that lasted approximately 90 minutes. We collected the following information from each participant: age, height, weight, living status, work status, education, duration since diagnosis, and other medical conditions.

The principal investigator (L.K.) was the moderator for the focus group and semistructured interviews. She holds a PhD degree in Rehabilitation and has previous experience in conducting focus groups and interviews. The moderator is considered an insider researcher since she is from the same culture as the participant and is familiar with the system at the hospital, which makes it easier to approach individuals and understand their perceptions and experiences [16]. To improve credibility of the data, the moderator ensured rephrasing, repeating and further explaining the questions when needed [17]. A relationship was not established with the participants before the start of the study, and the moderator is not involved in the participants' treatment plan. They were informed of the background of the moderator and the aims of the study. Two notetakers from the research team assisted the principal investigator during the focus group and interviews. These notes were compared with transcribed data to improve confirmability of the data [17].

The same topic guide was used for the focus group and semistructured interviews (Table 1). We performed a conceptual review of the literature to identify constructs relevant to knowledge of knee OA and its impact on health. Many key references were identified and included in the review [18–25].

The topic guide was developed after a consensus meeting was held to agree on key constructs relevant to the research question. The researchers ensured that all relevant constructs from the literature were included in the topic guide to establish construct validity [26]. Constructs included were knowledge of body changes with the disease, reasons for change in symptoms, management options to relief symptoms, and impact of knee OA on physical activity and participation. The topic guide was translated to Arabic by two researchers separately; a final version was approved by both researchers and shared with the research team.

The first focus group was a pilot for the topic guide. Results were reviewed by the research team, and there was a consensus that richness, variety, and relevance were demonstrated in participants' responses that serve the research aims. Therefore, this focus group was included in the subsequent main analysis, and no modification to the topic guide was performed.

Discussions were audiotaped and transcribed [27]. Sessions were conducted and transcribed in Arabic. Two separate researchers translated transcripts to English, and a third researcher compared and validated translations. The moderator as well as the notetakers confirmed the accuracy of the transcripts. Each participant was assigned a code number for data entry and quotations. Further analysis and interpretation were conducted in English.

### 2.4. Data Analysis

A pragmatic approach with an ontological approach of subtle realism was adopted during data analysis [28]. Framework analysis as described by Spencer et al. [29] was selected because it facilitates looking into participants' perspectives about an external existing reality. Constructs included in the topic guide were used as a framework to organize data for further indexing. The process includes five stages: (1) familiarization, (2) identifying thematic framework, (3) indexing, (4) charting, and (5) mapping and interpretation.

#### 2.4.1. Familiarization

This process involves getting familiar with emerging data and identifying key aspects, which was achieved when data were transcribed, translated, and verified by the research team.

#### 2.4.2. Identifying Thematic Framework

The thematic framework was established prior to data collection based on constructs emerging from the literature review. However, following the focus group, the framework proved to be adequate for indexing all data without the need for developing new themes.

#### 2.4.3. Indexing

Two research team members independently identified all participants' narratives relevant to a certain theme in the thematic framework and organized them under that theme.

#### 2.4.4. Charting

This included two stages: The first stage is a progression from indexing, where important ideas were grouped into categories by two researchers based on their interpretation. When interpretations differed, a third researcher was consulted and the issue was further discussed until a decision was made. Finally, emerging categories were grouped into classes. Three researchers performed this process separately and then approved one final version.

#### 2.4.5. Mapping and Interpretation

Themes were generated from the data set, and connections were made between participants and categories in an attempt to explain what was reported by them.

Final results were reported to the participants for feedback, and they agreed that these were their perspectives on the topic.

### 2.5. Trustworthiness of the Qualitative Enquiry

We previously reported strategies to achieve credibility, dependability, confirmability, and transferability during the preparation, data collection, and data analysis [17, 30].

### 2.6. Mapping to the ICF Framework

Three members of the research team mapped the data to the ICF framework independently. Identified items were confirmed by the research team, and any disagreements were resolved by discussions to reach consensus.

## 3. Results

Fourteen participants completed the study (age range from 43 to 77 years), and all of them lived with their families. Participants' characteristics are presented in Table 2.

The following themes and subthemes emerged.

### 3.1. Theme 1: Body Functions and Structures

#### 3.1.1. Physical Changes with Knee OA

Participants' main concern was pain in addition to stiffness, fatigue, swelling, crepitus, muscle tightness, and weakness. Other conditions complicated their situation such as asthma (ID 3) and back problems (IDs 7 and 10).

*I cannot go up and down the stairs or walk long distances. I have back pain which might have increased the problem (10)*

#### 3.1.2. Psychological Impact of Knee OA

Participants commonly expressed fear of pain as they linked it to worsening of knee OA. In addition, participants showed fear of flare-ups (*n* = 2), fear of increasing the damage in the knee (*n* = 2), fear of operative procedures (*n* = 1), fear of damaging the kidneys if analgesics were used (*n* = 1), and fear of falling (*n* = 1).

Participants were also frustrated by their condition; many activities were limited which increased their dependency on family members and restricted their participation in social events.

*… I am afraid that someone might bump into me and I would fall … If my daughter was not with me today, I would not have come (12)*

### 3.2. Theme 2: Activity Limitation and Participation Restriction

Reported activity limitations and participation restrictions and their ICF codes are presented in Table 3.

#### 3.2.1. Factors Influencing the Activities (Changes in Symptoms)

Most participants agreed their symptoms were variable and not constant. They referred the increase in symptoms to activities including from sitting to standing, ascending and descending stairs, house chores, twisting the knee, sleeping, sitting, walking long distances, walking uphill and downhill, standing for a long time, and while praying. Being tired or upset, cold weather, and gaining weight were also reported to increase knee OA symptoms.

*My knees hurt more when I am tired and if I stood for too long (1)*

Many participants believed an increase in symptoms suggests deterioration of knee OA and their knees will eventually get worse:

*I would not consider the increase in pain something normal. I know eventually things will get worse but at least now I am able to walk around compared to others who cannot (12)*

#### 3.2.2. Cultural Perspective to Activity Limitation and Participation Restriction

As a Muslim community, many of our participants reported limitation in praying; they would pray sitting on a chair rather than standing and heel-sitting. Moreover, house chores are mainly performed in Jordan by women, and since the majority of our participants were females, limitation in performing house chores was a common concern (*n* = 10).

*I do not move that much anymore and my housework is limited. I cannot clean high or low surfaces. I even pray sitting on a chair (12)*

In addition, all female participants except for one were housewives which could be a reflection of the culture in Jordan. The male participant was also unemployed (retired).

Therefore, there were no activity limitations or participation restrictions that are related to being employed.

#### 3.2.3. Social Perspective to Activity Limitation and Participation Restriction

Participants reported inability to attend social events because of their condition.

*I do not use the stairs anymore. So, I do not go to weddings or events if there was no elevator (5)*

Also, dependency on family members to perform certain activities such as house chores and going out was mentioned:

*I used to go out to get my things now I depend on my children to get them for me (11)*

### 3.3. Theme 3: Personal Factors

#### 3.3.1. Knowledge and Personal Interpretation of Disease Process

On the individual level, there was lack of knowledge of body changes with knee OA.

Participants' knowledge was limited to changes in structures; this included the cartilage (*n* = 10), muscles of the lower leg (*n* = 9), dryness (*n* = 7) or increase (*n* = 2) of the fluids inside the joint, and the patella (*n* = 2). Two participants did not know which structures were affected by knee OA.

One participant explained the causes of knee OA:

*the cartilage in the knee becomes dry either because of lack of movement, obesity, or genetic reasons,… (8)*

Despite this lack of knowledge, participants showed lack of interest in education about knee OA:

*I do not know why my pain increases. I do not care why it increases I only know there is pain (11)*

#### 3.3.2. Knowledge of Management Options to Relief Symptoms (Self-Management)

There was lack of knowledge of self-management options and dependency on physicians to manage knee OA:

*If I had a muscle spasm at home, I would book an appointment with my doctor (3)*

Participants described a number of strategies to manage their symptoms including creams and medications (*n* = 12), losing weight (*n* = 7), ice (*n* = 4), simple knee range of motion exercises (*n* = 3), elevation of the legs (*n* = 2), walking (*n* = 2), rest and pacing activities (*n* = 2), pillows under the knees (*n* = 1), knee brace (*n* = 1), physiotherapy (*n* = 1), and massaging (*n* = 1) or stretching (*n* = 1) a tight muscle.

Personal factors influenced the use of medication. Two participants did not use NSAIDs, one because of kidney problems and the other for fear of damaging the kidneys by medication. One participant refused to take medications because she did not want to expose her body to chemical substances. Another did not use medical creams because she hated their texture.

In addition, the possibility of gaining weight limited the use of some medications:

*The problem with medications is that they increase body weight. So, I do not take them unless I am really in pain (6)*

#### 3.3.3. Influence of Personal Factors on Activity and Participation

Participants knew gaining weight would increase their symptoms and decrease their physical activity whereas losing weight would improve their condition. However, there seemed to be a difficulty in changing behavior and maintaining a healthy lifestyle to lose weight:

*I know my body weight affects my knees. I lose weight but I gain it back again (1)*

Also, emotional and behavioral perceptions such as being upset or tired were reported to affect level of physical activity:

*If I feel energized and well I would move but if I am tired or upset, I feel sick and do not move at all (1)*

Lack of knowledge of how knee OA develops and its aggravating and relieving factors also caused fear and uncertainty that limited the participants' physical activity and participation:

*For three years now I do not climb stairs because I am afraid my knees would hurt. I live on the ground floor (3)*

Finally, support from family members encouraged the participants to go out and move:

*If my daughter was not with me today, I would not have come (12)*

### 3.4. Theme 4: Environmental Factors

Environmental factors influencing physical activity and participation were mapped to the ICF model and are presented in Table 4.

#### 3.4.1. Service Delivery Process

There is lack of a standardized management pathway with limited access to physiotherapy. The healthcare system in Jordan does not allow self-referral to physiotherapy. Therefore, the first contact point by our participants was their physicians. They were offered different management options including medications, surgery, and physiotherapy without any justification. Medications were prescribed to all participants whereas surgery was offered to two participants only. Physicians referred eleven participants to physiotherapy; however, it was not considered a priority; mainly, referral was either late or as an alternative to surgery.

*First, the Dr. told me I need surgery, but I did not want to do it. He told me to try physiotherapy (5)*

As for physiotherapy sessions, a predetermined package of 12 sessions that included hot or cold packs, electrotherapy, and exercise was prescribed to eleven participants.

Exercises were prescribed but inappropriately demonstrated by late prescription, lack of monitoring or feedback, and limited use of exercise leaflets (*n* = 2). In addition, the number of repetitions, type, and frequencies was also inappropriate. Strengthening exercises were rarely prescribed, and exercises were not progressed throughout the sessions. Most participants were advised to walk.

*I was told to pull my foot up and hold for 10* seconds *then relax. I was not provided with other exercises. My Dr. advised me to walk (3)*

#### 3.4.2. Ineffective Communication across the Care Pathway

Limited knowledge on knee OA is the consequence of lack of communication with physicians (IDs 2 and 6) and inadequate education offered by healthcare professionals. Participants mentioned that their physicians did not explain the underlying pathology; they mainly looked at X-rays and informed them if they were diagnosed with knee OA or not.

#### 3.4.3. Environmental Factors (Facilitators and Barriers)

Many environmental factors were reported to decrease physical activity and participation (barriers) including cold weather, stairs, and accessibility to services.

One participant reported waiting for two months for her physiotherapy sessions:

*The doctor referred me to physiotherapy, but it took me two months to start the sessions (8)*

On the other hand, facilitators to participation included tools to improve accessibility such as using cars, wheelchairs, and elevators.

*I do not go out much anymore … I always use the car. Even when I go to the mall I use a wheel chair …. (10)*

## 4. Discussion

Culture and lifestyle in the Middle East are different when compared to the developed world, which could influence the experience of people with knee OA. This is the first study to explore knowledge of knee OA and its impact on health in patients diagnosed with the condition living in the Middle East, particularly in Jordan. Limited knowledge of knee OA was reported, which in part is attributed to inappropriate service delivery process with poor communication with healthcare professionals and lack of education. Knee OA significantly limited physical activity and participation; however, this limitation was caused not only by their knees but also by the culture in Jordan. Mapping the results to the ICF framework allowed international comparisons emphasizing the impact of culture on health.

There was lack of understanding of body changes with knee OA; most limited the changes to affect body structures only and none mentioned changes to body functions as in muscle weakness. Knee OA is known in Arabic as “roughness of the knee” which might explain why most considered knee OA as dryness of the liquid inside the joint and increased friction. Little was known on how it develops, risk factors, and prognosis. Limited knowledge of the pathology of knee OA was previously reported in the United Kingdom [31]. Moreover, perspectives towards knee OA were similar worldwide; it was perceived as an “inevitable” condition that would deteriorate with age [4].

Our participants' experience of symptoms with knee OA was comparable to those of other populations worldwide including pain, stiffness, fatigue, swelling, muscle weakness, crepitus, and fear [4]. Fear included fear of falling, fear of deterioration of OA, and fear of becoming a burden [4]. This fear would limit activities and participation and increase risk of disability [32]. Therefore, it is essential to overcome fear of pain and falling which could be established through education and self-management programs [33]. Our participants depended on their families to overcome these fears. However, similar to other studies, there was a sense of frustration caused by loss of independence [34, 35].

Our participants were not aware of appropriate self-management options or behavioral modification techniques to manage their symptoms. Several studies reported patients with knee OA lacked the knowledge that would enable them to manage their pain [31, 35–37]. Comparable with other studies, the use of medication was the first option [38, 39]. One would expect the participants who were referred to physiotherapy to use exercises more often to decrease their symptoms and improve function. However, lack of education on the importance of exercise, inappropriate service delivery process demonstrated by late referrals to physiotherapy, and limited and inappropriate exercise prescription directed the participants to use medications to relieve their pain.

Our participants also referred their limited knowledge to insufficient information provided by healthcare professionals. It is worth noting that many of our participants had basic school education, so their ability to read and seek information by themselves was limited. Lack of education resulted in more pain, depression, and lack of coping strategies [40]. Therefore, international guidelines recommend providing education individualised to each participant including knowledge of OA, pacing activities, exercise, weight loss, and assistive technology [20].

Exploring the experiences of people diagnosed with knee OA worldwide, mainly in developed countries, demonstrated that activity limitations were similar across populations including walking, standing, sitting, and ascending and descending stairs [4, 31, 36, 41]. Our participants had similar activity limitations in addition to restrictions in religious activities. Xie et al. [41] explored the impact of knee OA on different cultures in Singapore and reported comparable results as they have a large Muslim population.

A few of our participants reported limitation in walking long distances (*n* = 6), and none reported limitation in activities such as getting on or off a bus, travelling, driving, or activities needed for a certain job. Those activities were restricted by knee OA in Singapore [41]. Also, limitation in work performance was reported by different populations [4]. However, Jordan has a different culture; most female participants in our study are housewives and the male participant is retired, and Jordan has many hills and valleys limiting walking long distances. From the social perspective, families are accustomed to attending many social events. Our participants reported restrictions in socializing where they would depend on family members to help them.

Environmental facilitators included using cars, elevators, and wheelchairs. On the other hand, uneven ground, cold weather, and service accessibility difficulties adversely affected the participants' experience. Xie et al. [41] was the only study to explore environmental factors affecting participants from Singapore and reported similar results.

This is the first study to explore knowledge of knee OA and its impact on health in the Middle East and to compare the results with developed countries. Using a triangulation method enriched the data. Mapping our results to the ICF framework allowed comparability with other studies in addition to exploring environmental and personal factors which was limited in previous studies. Furthermore, the principal investigator conducted the focus group and interviews which allowed close observation of participants' responses and elicited more responses based on her experience with knee OA. Nonetheless, consensus meetings were held by the research team to interpret the data to avoid imposing previous knowledge on emerging data [42].

Study limitations include having access to one local hospital; therefore, a convenient sampling method was used instead of the ideal purposive method. Although this is a central hospital in the capital Amman, the results might not be generalised to the Jordanian population. Moreover, the majority of participants were middle-aged unemployed females. We aimed to conduct gender-specific focus groups to respond to cultural sensitivity and take into consideration that activities and participation might be gender-specific. However, we were able to recruit one male participant only, which might have biased our results. This could be explained by the culture as men are perceived as the main providers for their homes and do not have time to participate in research whereas older women are mainly housewives and have time. Therefore, future studies should explore the perspectives of a larger sample of both genders with a wider educational background and work experiences and in different settings in Jordan.

## 5. Conclusions

The results demonstrate that the effect of knee OA varies among different cultures and highlight the role of healthcare professionals worldwide in understanding the impact of culture on health. They also increase the awareness of healthcare professionals in Jordan on the limitations in delivered services and the importance of education.

## Figures and Tables

**Table 1:** Topic guide for the focus group and the semistructured interviews.

(i) Knowledge of knee osteoarthritis (OA) (a) Structures affected by knee OA (b) Reasons for increase in symptoms (c) Management options to relief symptoms (ii) Management of knee OA (a) Options offered by physician (iii) Impact of OA (a) Chief complaint (b) Variability of symptoms (c) Impact on activities of daily living (d) Impact on participation

**Table 2:** Demographic data of the participants (mean (SD)).

Age (years)	60.21 (9.4)		

Gender (*n*, %)	F (*n* = 13, 92.9%)	M (*n* = 1, 7.1%)	

Weight (kg)	85.5 (13.68)		

Height (cm)	166 (10.18)		

Time since diagnosis (years)	6.51 (8.13)		

Education (*n*, %)	High school (*n* = 6, 42.9%) Diploma (*n* = 3, 21.4%) Illiterate (*n* = 2, 14.1%)		8^th^ grade (*n* = 1, 7.1%) 6^th^ grade (*n* = 1, 7.1%) BSc. (*n* = 1, 7.1%)

Occupation (*n*, %)	House wife (*n* = 12, 85.7%) Retired (*n* = 1, 7.1%)		Teacher (*n* = 1, 7.1%)

Other medical conditions (*n*, %)	Hypertension (*n* = 7, 50%) Diabetes (*n* = 3, 21.4%) Heart disease (*n* = 2, 14.3%) Degenerative disc disease (*n* = 1, 7.1%)		Kidney problems (*n* = 1, 7.1%) Inner ear problem (*n* = 1, 7.1%) None (*n* = 3, 21.4%)

Kellgren and Lawrence scale (*n*, %)	Unilateral	Grade 3	*n* = 2, 14.3%
	Bilateral	Grade 2	*n* = 6, 42.9%
		Grade 3	*n* = 4, 28.6%
		Grades 2 & 3	*n* = 2, 14.3%

**Table 3:** The reported activity limitations and participation restrictions and their ICF codes.

Activity limitations	ICF code and title	Activity limitations	ICF code and title
Sit to stand (*n* = 11)	d4103 sitting	Sleeping (*n* = 2)	d4150 maintaining a lying position, b1343 quality of sleep

Ascending and descending stairs (*n* = 11)	d4551 climbing	Pain when carrying heavy objects (*n* = 2)	d4302 carrying in the arms

House work (*n* = 10)	d6400 washing and drying clothes and garments d6401 cleaning cooking area and utensils d6402 cleaning living area	Pacing (*n* = 2)	d2101 undertaking a complex task

Praying at home (*n* = 6)	d9308 religion and spirituality, other specified	Moving around at home (*n* = 2)	d4600 moving around within the home

Walking long distances (*n* = 6)	d4501 walking long distances	Difficult to pick up anything from the ground or even the lower kitchen cupboards (*n* = 2)	d4101 squatting, d4102 kneeling, d4105 bending

Walking short distances (*n* = 3)	d4500 walking short distances	Walking on uneven surfaces (*n* = 1)	d4502 walking on different surfaces

Standing (*n* = 3)	d4104 standing	Standing for a long time (*n* = 1)	d4154 maintaining a standing position

Sitting for a long time (*n* = 1)	d4153 maintaining a sitting position		

*Participation restrictions*		*ICF code and title*	
Do not go out of their homes as before for errands, shopping, or social events (*n* = 11)		d4602 moving around outside the home and other buildings d6200 shopping d9205 socializing	

**Table 4:** The environmental factors affecting the participants and their ICF codes.

Environmental factors	ICF code and title
Support from family members (facilitator) (*n* = 4)	E310 immediate family
Using a car (facilitator) (*n* = 1) Using a wheelchair (facilitator) (*n* = 1)	e1 200 general products and technology for personal indoor and outdoor mobility and transportation
Elevators (facilitator) (*n* = 1)	e1501 design, construction, and building products and technology for gaining access to facilities inside buildings for public use
Lack of education (barrier) (*n* = 10)	e450 individual attitudes of health professionals e5850 education and training services
Cold weather (barrier) (*n* = 1)	e2 250 temperature

## Data Availability

The chart of indexing and categorizing emerging themes in the pre-developed framework and the identified classes table are available upon request from the corresponding author.

## Supplementary Materials

Supplementary Materials Appendix 1: Standards for Reporting Qualitative Research (SRQR).
